# MOF-Derived Nitrogen-Doped Porous Carbon Polyhedrons/Carbon Nanotubes Nanocomposite for High-Performance Lithium–Sulfur Batteries

**DOI:** 10.3390/nano13172416

**Published:** 2023-08-25

**Authors:** Jun Chen, Yuanjiang Yang, Sheng Yu, Yi Zhang, Jiwei Hou, Nengfei Yu, Baizeng Fang

**Affiliations:** 1 College of Electrical Engineering and Control Science, Nanjing Tech University, Nanjing 211816, China; 2 School of Energy Sciences and Engineering, Nanjing Tech University, Nanjing 211816, China; 3 Department of Chemistry, Washington State University, Pullman, WA 99164, USA; 4 Department of Energy Storage Science and Technology, University of Science and Technology Beijing, Beijing 100083, China; baizengfang@163.com

**Keywords:** lithium–sulfur batteries, carbon nanotubes, metal–organic framework, shuttling effect, chemical immobilization

## Abstract

Nanocomposites that combine porous materials and a continuous conductive skeleton as a sulfur host can improve the performance of lithium–sulfur (Li-S) batteries. Herein, carbon nanotubes (CNTs) anchoring small-size (~40 nm) N-doped porous carbon polyhedrons (S-NCPs/CNTs) are designed and synthesized via annealing the precursor of zeolitic imidazolate framework-8 grown in situ on CNTs (ZIF-8/CNTs). In the nanocomposite, the S-NCPs serve as an efficient host for immobilizing polysulfides through physical adsorption and chemical bonding, while the interleaved CNT networks offer an efficient charge transport environment. Moreover, the S-NCP/CNT composite with great features of a large specific surface area, high pore volume, and short electronic/ion diffusion depth not only demonstrates a high trapping capacity for soluble lithium polysulfides but also offers an efficient charge/mass transport environment, and an effective buffering of volume changes during charge and discharge. As a result, the Li-S batteries based on a S/S-NCP/CNT cathode deliver a high initial capacity of 1213.8 mAh g^−1^ at a current rate of 0.2 C and a substantial capacity of 1114.2 mAh g^−1^ after 100 cycles, corresponding to a high-capacity retention of 91.7%. This approach provides a practical research direction for the design of MOF-derived carbon materials in the application of high-performance Li–S batteries.

## 1. Introduction

Compared with the currently used Li-ion battery intercalation electrode materials, the S element has overwhelming advantages with highly crustal abundance, low production cost, and environmental benignity [1,2,3], especially the high theoretical specific energy of 1675 mAh g^−1^, over three times higher than that of traditional intercalation electrode materials, making Li-S batteries potential candidates as power sources for next-generation electric vehicles, grid-scale stationary energy storage systems, and other electrical devices. For Li-S batteries, the S_8_ ring molecules first react with Li^+^ to form long-chain Li_2_S_6_. Then, Li_2_S_6_ transforms into short-chain Li_2_S_4_ through successive cleavage of S-S bonds [4]. Despite the existence of these great advantages, large-scale commercial application of Li-S batteries still faces various challenges: (1) the poor intrinsic conductivity of both S (5 × 10^−30^ S cm^−1^) and Li_2_S (10^−13^ S cm^−1^) leads to the low utilization of active materials and poor rate capability [5]; (2) about 80% of volume changes caused by the full lithiation and delithiation during discharge and charge processes induce the degradation of electrode structure and reduction of cycling performance [6]; and (3) the serve “shuttle effect”. When Li-S batteries’ organic liquid electrolytes are used, the higher-order lithium polysulfide intermediates (Li_2_S_x_ 4 ≤ x ≤ 8) form during cycling. These polysulfide intermediates migrate from the cathode to the anode, under a gradient concentration, where they react with metallic lithium to form shorter polysulfides, and vice versa, activating the so-called “shuttle effect” which leads to low Coulombic efficiency and rapid capacity fade [7].

Over the past few decades, great efforts to improve the electrical conductivity and buffer the volume changes for the S cathode as well as suppress the shuttle effect of soluble polysulfides during cycling, have been focused on the S-based composite cathode with these advanced features of high conductivity, efficient ion pathway, and sufficient void space to accommodate the volume changes [8]. Carbon-based materials have been widely concerned and explored as S hosts for Li-S batteries owing to their features of a large specific surface area, high electric conductivity, and tunable porous structures [9]. To date, various structural carbon-based materials, such as activated carbon [10], graphene [11,12], CNTs [13], micro-/meso-porous carbon [14], carbon hollow spheres [15], hierarchically porous carbon [16], and carbon sponge [17,18,19], etc., have been synthesized and employed as hosts of S cathodes. However, the nonpolar carbon materials poorly interact with the polar polysulfides, which makes it hard to suppress the shuttle effect of the soluble polysulfides and reduce S loss [20,21]. Generally, introducing polar materials in carbon materials is an effective strategy to strengthen the chemical interactions between carbons and polysulfides, inhibiting the migration of polysulfides [22].

Metal–organic framework (MOF) materials are a new type of porous organic–inorganic hybrid materials that have many advantages. First, MOFs are characterized by a high specific surface area which allows for significant sulfur loading, increasing the overall energy density of the Li-S batteries. Second, MOFs are of well-defined porosity which can also accommodate the diffusion of sulfur, leading to improved electrochemical performance. Third, MOFs can be designed with tunable pore sizes which enable the optimization of Li^+^ transport pathways, leading to improved rate performance. Thus, MOFs have prospective applications in Li-S batteries [23]. Moreover, MOFs can adsorb Li_2_S_2_ onto their porous structures. The high surface area and porosity of MOFs provide sites where Li_2_S_2_ molecules can physically adsorb or even be encapsulated within the pores. This interaction can help mitigate the shuttling effect of intermediate sulfur species, reducing their migration within the battery and improving overall battery performance [24]. However, MOF materials still face the problem that their electrical conductivities are not high enough due to their insulation. Annealing can convert MOFs to porous carbons, improving the overall electrical conductivity of the composite. Building composite structures with MOF-derived carbons dispersing uniformly on the continuous conductive skeletons (e.g., CNTs, graphene foam, and carbon nanofiber matrix) is a popular approach to further enhancing the electrical conductivity [25,26,27]. Therefore, the rational design and synthesis of advanced hosts for S cathodes are still critical issues to fully realize high-performance Li-S batteries.

Herein, we present the in situ growth of small-size zeolitic imidazolate framework-8 (ZIF-8) nanoparticles on CNTs, resulting in a composite of CNTs anchoring small-size (~40 nm) N-doped porous carbon polyhedrons (S-NCPs/CNTs) after thermal treatment. In the composite, the S-NCPs serve as an efficient host for trapping polysulfides through physical adsorption and chemical bonding, while the interleaved CNT networks offer an efficient charge transport environment. Moreover, the S-NCP/CNT nanocomposite with great features of a large specific surface area, high pore volume as well a short electronic/ion diffusion path serves as an efficient host for the S cathode (S/S-NCPs/CNTs), which not only demonstrates high trapping capacity for soluble lithium polysulfides but also offers an efficient charge/mass transport environment, and an effective buffering of volume changes during charge and discharge. The Li-S batteries based on the S/S-NCP/CNT cathode deliver an initial capacity of 1017.1 mAh g^−1^ at a current rate of 1 C and a substantial capacity of 913.8 mAh g^−1^ after 500 cycles, corresponding to a low capacity decay of only 0.02% per cycle.

## 2. Experimental

### 2.1. Materials

Polyvinylpyrrolidone (PVP, MW: ca. 27,300, Aladdin, Shanghai, China), 2-methylimidazole (2-MeIm, 98%, Aladdin), zinc nitrate hexahydrate (99%, Aladdin), carbon nanotubes (CNTs, >95%, Aladdin), polyvinylidene (PVDF, Aladdin), fluoride methanol (>99.5%, Macklin, Shanghai, China), N-methyl-pyrrolidone (NMP, AR, Aladdin), and concentrated nitric acid (GR, Aladdin) were used as received without further purification. The lithium–sulfur electrolyte (0.5 M LiCF_3_SO_3_, 0.5 M LiNO_3_ in 1,2-dimethoxyethane (DME): 1,3-dioxolane (DOL) =1:1 vol%) was purchased from DodoChem Co., Ltd., Zhuhai, China.

### 2.2. Synthesis of S-NCP/CNT Nanocomposite

The S-NCP/CNT nanocomposites were constructed using the CNTs anchoring small-size (~10 nm) N-doped porous carbon polyhedrons via annealing the precursor of small-size zeolitic imidazolate framework-8 grown in situ on CNTs (S-ZIF-8/CNTs). First, the S-ZIF-8/CNT precursors were synthesized by the solvothermal method. Typically, 150 mg of acidified CNTs and 3.0 mmol of 2-MeIm were successively added into 200 mL of methanol containing 100 mg of PVP under vigorous stirring to form homogeneous ink. Subsequently, 1.5 mmol of Zn(NO_3_)_2_·6H_2_O was added to the above methanol solution and stirred for 15 min and then the resulting mixture was transferred into a Teflon-line autoclave and heated to 90 °C for 6 h. After the solvothermal reaction, the obtained precipitates, S-ZIF-8/CNT composites, were collected by centrifugation, washed three times with methanol and deionized water, and then dried in vacuum at 60 °C overnight. Finally, annealing the as-obtained S-ZIF-8/CNTs precursors under an Ar atmosphere in a tube furnace at 920 °C for 3 h with a ramp rate of 2 °C min^−1^ generated S-NCP/CNT nanocomposites.

### 2.3. Fabrication of S/S-NCP/CNT Nanocomposite

A simple method of solution infiltration and heat treatment was applied to controllably load sulfur into S-NCP/CNT nanocomposite, generating sulfur-containing nanocomposite S/S-NCPs/CNTs. Before loading sulfur, the S-NCP/CNT nanocomposite was heated at 120 °C maintained for 3 h for activation treatment. In a typical fabrication of S/S-NCPs/CNTs, the as-activated S-NCP/CNT nanocomposite was immersed in sulfur/CS_2_ solution (25 mg/mL) for 72 h, in which the weight ratio of S-NCPs/CNTs and sulfur was controlled at 2 to 8. Then, the mixture was dried at 40 °C. Next, the obtained sulfur-containing nanocomposites were heated at 155 °C in a sealed autoclave for 12 h, generating the S/S-NCP/CNT nanocomposite.

### 2.4. Materials Characterization

X-ray diffraction (XRD) patterns were recorded in an X-ray diffractometer (SmartLab3KW, Tokyo, Japan) with Cu-Ka radiation (λ = 0.1540 nm) at 40 kV, 40 mA and scanning rate of 10°/min in a 2θ range of 10–80°. Scanning electron microscopy (SEM) and transmission electron microscopy (TEM) experiments were performed on a Phenom ProX Scanning Electron Mircoscope and a JEM1200EX high-resolution transmission electron microscope, respectively. Thermal gravimetric analysis (TGA) and differential scanning calorimetry (DSC) were conducted with a TGA thermogravimetric analyzer in argon at a scan rate of 10 °C/min from room temperature to 800 °C. The Brunauer–Emmett–Teller (BET) surface area was determined through nitrogen adsorption isotherms using an Autosorb iQ3 autosorb automated gas sorption system.

### 2.5. Electrochemical Measurements

Electrochemical measurements were carried out in CR2025 coin-type cells using sulfur cathodes, Li foil anodes, commercial PP, separators, and 40 μL of electrolyte (0.5 M LiCF_3_SO_3_, 0.5 M LiNO_3_ in DME: DOL = 1:1 vol%). The sulfur cathodes were prepared by mixing NC/CNT/S composites, carbon black, and PVDF with a weight ratio of 8:1:1, dispersed in NMP to form the equal slurry, and coated on an Al foil. The S mass loading is about 1.27 mg cm^−2^. Thus, the ratio of sulfur/electrolyte is 0.025 mg µL^−1^. The cyclic voltammetry (CV) measurements were carried out using a CHI760e electrochemical workstation at a scan rate of 0.1 mV s^−1^ with a voltage range of 1.7–2.8 V. Electrochemical impedance spectroscopy (EIS) was performed on a PARSTAT 2273 in a frequency range from 10^−1^–10^5^ Hz. The galvanostatic charge/discharge tests were conducted on LAND CT2001A in a voltage window between 1.7 and 2.8 V at 0.1–5 C rates (1 C = 1675 mA g^−1^).

## 3. Results and Discussion

**Figure 1:**
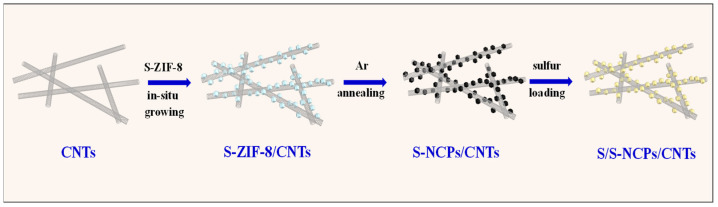
Schematic illustration of synthesis process for fabricating nanocomposite of S/S-NCPs/CNTs.

The fabrication procedure of S/S-NCPs/CNTs is illustrated in Figure 1. The precursor of S-ZIF-8/CNTs was first obtained via small-size ZIF-8 particles growing in situ on CNTs. Then, the as-obtained precursor of S-ZIF-8/CNTs was annealed at 920 °C under Ar atmosphere, and generated S-NCPs/CNTs. During carbonization at a high temperature, the N-containing methylimidazole ligand changed qualitatively to form rich N-doped graphitized carbon [28]. Additionally, the evaporation of Zn initiates the formation of microporous carbon, significantly increasing the available surface area [27]. Finally, a method combining solution infiltration and heat treatment was used to controllably load sulfur into the S-NCP/CNT nanocomposite, generating sulfur-containing nanocomposites of S/S-NCPs/CNTs.

**Figure 2:**
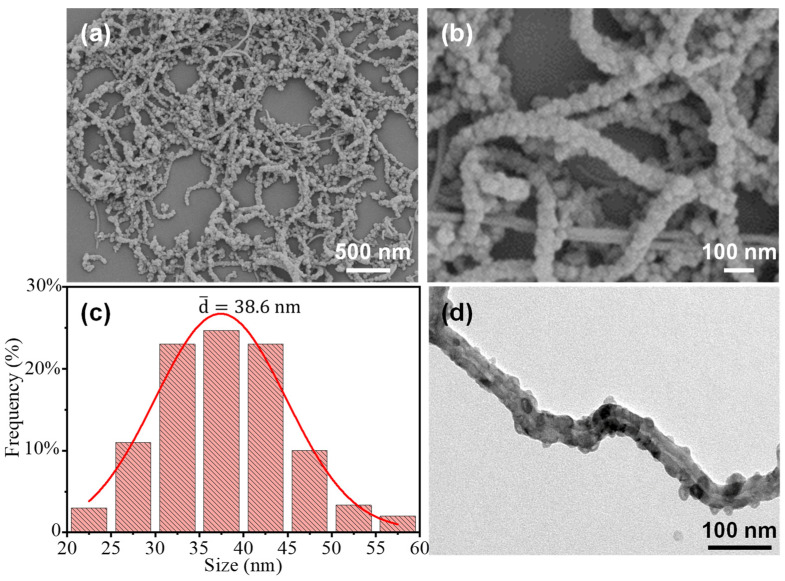
(**a**) Low-magnification, (**b**) high-magnification SEM images, and (**c**) size distribution of the NCP particles in the S-NCPs/CNTs. (**d**) TEM image of the S-NCPs/CNTs.

The morphologies of the samples were characterized using SEM and TEM techniques. Figure 2a,b show that the S-NCPs/CNTs exhibit a core–shell structure, in which the outer shell is transformed from S-ZIF-8 to S-NCPs due to the high-temperature carbonization. The outer shell of S-NCPs owns small particles with sizes ranging from 20 to 60 nm (average particle of 38.6 nm) (Figure 2c), which could be caused by massive loss from 2-methylimidazole ligand gasification during the annealing process [27,29]. The TEM image (Figure 2d) not only further indicates that the S-NCPs/CNTs feature the core–shell structure with the CNT core encapsulated by outer nanoparticles S-NCPs, but also shows that the S-NCP nanoparticles are firmly anchored to CNTs.

**Figure 3:**
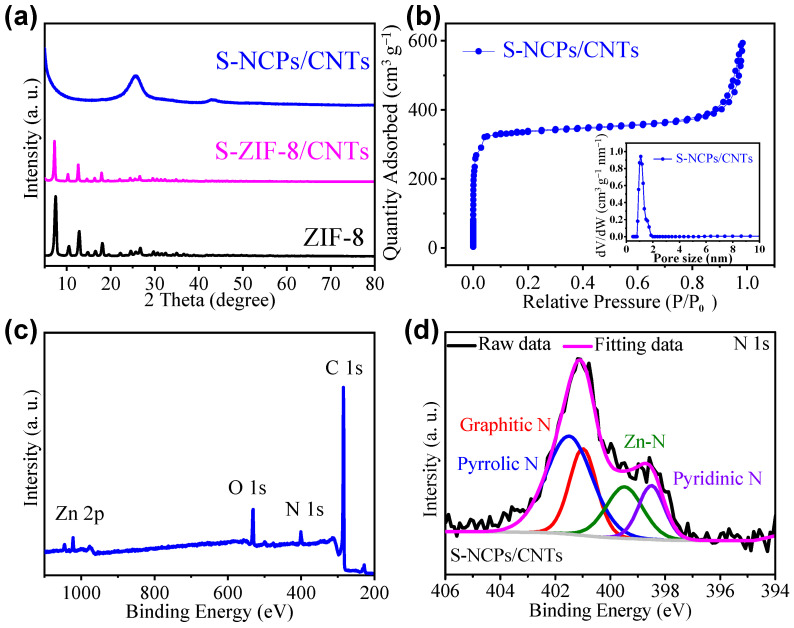
(**a**) XRD patterns of ZIF-8, S-ZIF-8/CNTs and S-NCPs/CNTs. (**b**) Nitrogen adsorption–desorption isotherms for S-NCPs/CNTs and the pore-size distribution for S-NCPs/CNTs. (**c**) Full XPS spectrum of S-NCPs/CNTs. (**d**) XPS high-resolution of N 1s.

The crystal structure of the samples before and after annealing was characterized using the XRD technique. There is only the appearance of characteristic peaks of ZIF-8 crystalline (JCPDS No. 62-1030) [30] in the S-ZIF-8/CNTs (Figure 3a), confirming that the ZIF-8 nanoparticles were successfully prepared. After annealing, the ZIF-8 nanoparticles were fully transformed to NCPs, which can be confirmed via the typical diffraction peaks of graphitized carbons in the XRD pattern of the S-NCPs/CNTs. The surface area and porous structure of the S-NCPs/CNTs were investigated by isotherm nitrogen adsorption–desorption measurement. Figure 3b indicates that S-NCPs/CNTs present the type-I isotherms with the unique feature of sharp uptakes at a low relative pressure (P/P_0_ < 0.1), suggesting the presence of a large volume of micropores. The micropore size distribution ranges from 1 to 2 nm. The surface area and the pore volume of the S-NCPs/CNTs were measured to be 1135.2 m^2^ g^−1^ and 0.47 cm^3^ g^−1^, respectively. The large surface area and pore volume are not only beneficial to accommodate volume change of the active material S caused by full lithiation and delithiation during discharge and charge processes but also can provide the trapping capability of carbon-based materials to sulfur species; hence, it is expected that the S-NCPs/CNTs can possess excellent capabilities of immobilizing sulfur and trapping polysulfides for effectively reducing the sulfur loss and inhibiting the polysulfide migration. The XPS technique was applied in the identification of the chemical composition and electronic states of the S-NCPs/CNTs. As shown in Figure 3c, the full spectrum clearly reveals the presence of C, N, and O, and a small amount of un-gasified Zn on the surface of the derivatives. The electronic states of N in the S-NCPs/CNTs are clearly shown in Figure 3d. Four deconvoluted peaks at 398.5, 399.3, 401.2, and 401.5 eV correspond to pyridinic N, Zn-N, graphitic N, and pyrrolic N [31], respectively. All these four types of N species are helpful in strengthening the polysulfide bonding through different approaches, either to form strong chemisorption of polysulfides by the Li-N interactions or to improve the binding energy between polar polysulfides and nonpolar carbon [32].

**Figure 4:**
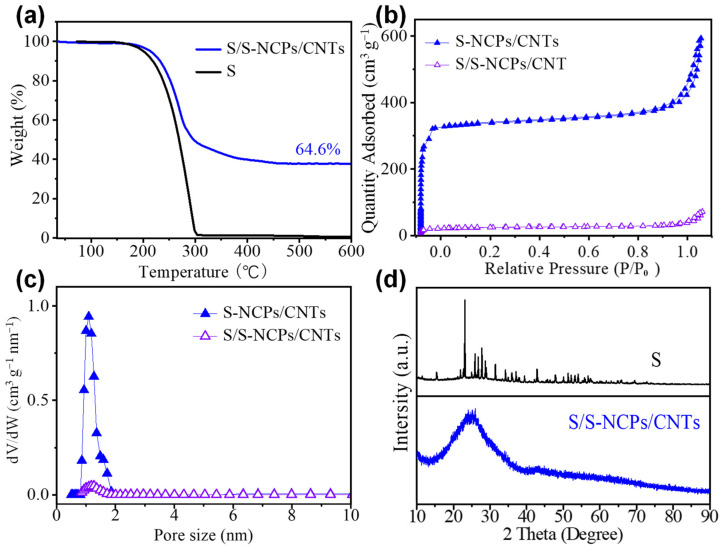
(**a**) TGA curves of S/S-NCPs/CNTs and pure S under Ar until 600 °C. (**b**) Nitrogen adsorption–desorption isotherms for the S/S-NCPs/CNTs. (**c**) The pore-size distribution for S/S-NCPs/CNTs. (**d**) XRD patterns of crystalline S, S/S-NCPs/CNTs.

A method of combining solution infiltration and heat treatment was used to load sulfur into S-NCPs/CNTs, generating sulfur-containing nanocomposite S/S-NCPs/CNTs. The sulfur content in the composite was measured using TGA analysis in an N_2_ atmosphere with temperatures ranging from 35 to 600 °C, as demonstrated in Figure 4a. As a comparison, the pristine sulfur was also underwent TGA analysis, which exhibited a sharp process of weight loss starting around 155 °C and finishing complete weight loss before 305 °C. In contrast, the S/S-NCP/CNT composite shows a total weight loss of up to 500 °C. This hysteresis implies that there are some physical and chemical adsorption processes between elemental S and N-doped porous carbon materials leading to the weight loss of S in the composite being more difficult. The TGA curves state clearly that the S content in the S/S-NCPs/CNTs is ca. 64.6 wt% [33]. The sulfur distribution was further determined by isotherm nitrogen adsorption–desorption technology, as shown in Figure 4b. The surface area decreases were from ca. 1135.2 m^2^ g^−1^ of the S-NCPs/CNTs to 80.4 m^2^ g^−1^ of the S/S-NCPs/CNTs, according to the BET test. Additionally, the micropore volume also reduces dramatically from 0.47 cm^3^ g^−1^ of the S-NCPs/CNTs to 0.02 cm^3^ g^−1^ of the S/S-NCPs/CNTs, based on the change in pore size distribution before and after sulfur loading (Figure 4c). These results indicate that almost all sulfur is filled into the micropores of the S-NCPs/CNTs [34]. Furthermore, no characteristic peaks of crystalline sulfur are observed in the XRD pattern of the S/S-NCPs/CNTs (Figure 4d), also confirming that the elemental sulfur in the form of an amorphous state was loaded into the micropores [35].

**Figure 5:**
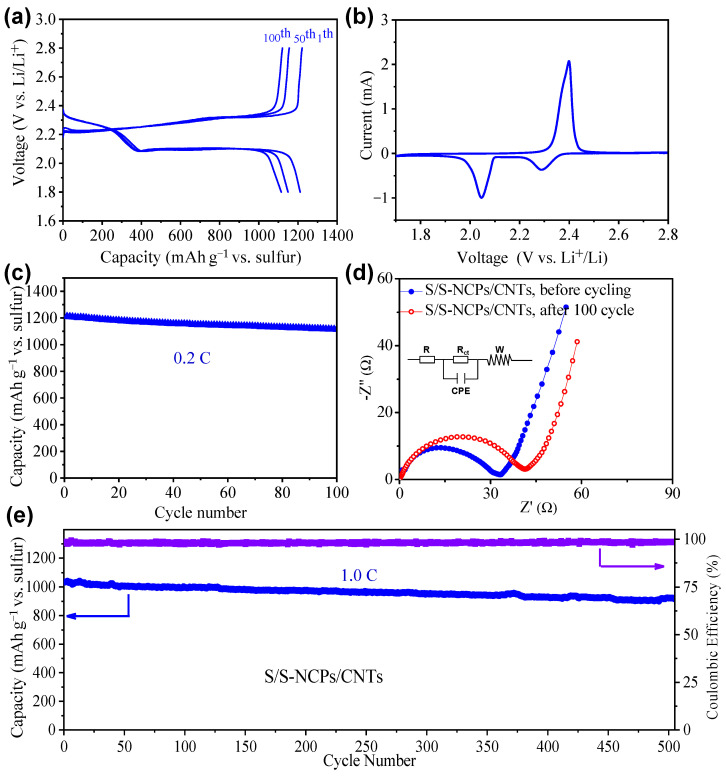
(**a**) Galvanostatic charge/discharge profiles of the S/S-NCPs/CNTs at 0.2 C. (**b**) CV curves of the S/S-NCPs/CNTs at a scan rate of 0.1 mV s^−1^. (**c**) Cycle performances of the S/S-NCPs/CNTs at 0.2 C. (**d**) The Nyquist plots of the cell with the S/S-NCPs/CNTs before cycling and after 100 cycles. (**e**) Cycling performance of the S/S-NCPs/CNTs at 1.0 C.

To evaluate the practical applicability of the S/S-NCPs/CNTs as an effective cathode for Li-S batteries, a series of electrochemical assessments were carried out through assembling coin cells with metallic lithium as the anode. The capacities of all Li-S cells were obtained on the basis of the mass of sulfur. The galvanostatic charge/discharge profiles of the S/S-NCPs/CNTs recorded at a current rate of 0.2 C (1 C = 1675 mA g^−1^) are presented in Figure 5a, in which a charge plateau and two discharge plateaus can be apparently observed. For the discharge process, a short upper discharge plateau around 2.3 V and a lower discharge plateau around 2.1 V correspond to the reduction from solid elemental sulfur to soluble long-chain polysulfides (Li_2_S_4–8_), and further reduction from Li_2_S_4–8_ to insoluble products Li_2_S_2_/Li_2_S, respectively. In the charge process, a single long charge plateau from 2.2 to 2.4 V indicates the conversion of Li_2_S_2_/Li_2_S into elemental sulfur and lithium [36].

The CV curves of the cathode at a scan rate of 0.1 mV s^−1^ are shown in Figure 5b, where two cathodic reduction peaks and an anodic oxidation peak are observed, which is consistent with the two discharge plateaus and a charge plateau in the galvanostatic charge/discharge profiles [37]. Figure 5c shows the cycling performance of the S/S-NCP/CNT cathode at a current rate of 0.2 C. The S/S-NCPs/CNTs exhibited a slight reduction in the discharge capacity from 1213.8 to 1114.2 mA h g^−1^, keeping a high capacity retention of 91.7% [38]. This is attributed to the fact that the S-NCPs/CNTs possess a high conductivity, which improves the utilization of insulate sulfur and kinetics of polysulfide redox reactions by reducing the charge-transfer resistance of the electrochemical reaction.

The electrode kinetics were studied using EIS technology. Figure 5d shows the Nyquist plots of the cells with the S/S-NCPs/CNTs before cycling and after 100 cycles, which are composed of a depressed semicircle and a linear section in the high- and low-frequency regions, respectively. The corresponding equivalent circuit is shown in the insert of Figure 5d. It includes a resistor representing the solution resistance, a charge-transfer resistance (*R*_ct_), a constant phase element (CPE) representing the double-layer capacitance, and a Warburg element (W) accounting for diffusion processes [39,40]. The charge-transfer resistance of the S/S-NCPs/CNTs collected before cycling is 32.6 Ω. After 100 cycles, the charge-transfer resistance of the S/S-NCPs/CNTs increases by only 8.6 Ω to 41.2 Ω [41]. This further confirms that the sulfur-loaded small-size NCPs with high conductivity can significantly accelerate the kinetics of polysulfide redox reaction by reducing the charge-transfer resistance of the electrochemical reaction [42]. We further tested the cycling performance of the S/S-NCP/CNT cathode at a high rate (1.0 C). As shown in Figure 5e, the high rate-cycling performance of the S/S-NCP/CNT cathode is also good.

## 4. Conclusions

In summary, we demonstrated an advanced S/S-NCP/CNT cathode in the form of CNTs anchoring small-size NCPs as efficient sulfur hosts. In the S/S-NCP/CNT cathode, the S-NCPs with great features of a large specific surface area and pore volume as well as short electronic/ion diffusion paths not only demonstrate a high trapping capacity for soluble lithium polysulfides but also effectively buffer the volume changes during the charge and discharge, while the interleaved CNT networks offer an efficient charge transport environment. As a result, the Li-S battery based on the S/S-NCP/CNT cathode delivers a high initial capacity of 1213.8 mAh g^−1^ at a current rate of 0.2 C and a substantial capacity of 1114.2 mAh g^−1^ after 100 cycles, corresponding to a high capacity retention of 91.7%. Moreover, the Li-S battery based on the S/S-NCP/CNT cathode possesses a long lifespan (500 cycles) at 1.0 C which is close to that of high-energy-density LIBs, showing great potential in the energy storage market. The strategy demonstrated in this work provides an effective approach for the rational design of high-performance sulfur cathodes and can be extended to other fields.

## Data Availability

Data will be available upon request from the corresponding authors.
